# The Contextual Cat: Human–Animal Relations and Social Meaning in Anglo-Saxon England

**DOI:** 10.1007/s10816-014-9208-9

**Published:** 2014-04-24

**Authors:** Kristopher Poole

**Affiliations:** Department of Archaeology, University of Nottingham, Humanities Building, University Park, Nottingham, NG7 2RD UK

**Keywords:** Agency, Performativity, Zooarchaeology, Animals, Anglo-Saxon England, Classification

## Abstract

The growing popularity of relational approaches to agency amongst archaeologists has led to increased attention on the specific contexts of interaction between humans and their material worlds. Within such viewpoints, non-humans are perceived as agents in their own right and placed on an equal footing with humans, with both acting to generate social categories in past cultures. However, to date, the focus of these interpretative models has been overwhelmingly directed towards inanimate objects. Animals are generally absent from these discussions, despite their ubiquity in past societies and the frequently central roles they held within daily lives and social relations. Moreover, living animals are set apart from material culture because, like humans, they are usually aware of their environs and are capable of physically responding to them. This ability to ‘act back’ would have made human–animal interactions extremely dynamic and thus offers different conceptual challenges to archaeologists than when faced with objects. This paper demonstrates that the notion of performativity, combined with understanding of animals themselves, can help to comprehend these relations. It does so by focusing on one particular species, the domestic cat, in relation to Anglo-Saxon England. The characteristics and behaviour of these animals affected the ways in which humans perceived and interacted with them, so that just one individual cat could be categorised in a range of different ways. The classification of animals was thus just as fluid, if not more so, as that of objects and highlights the need to incorporate the former into reconstructions of the social in archaeological research.

## Introduction

For much of the time in which agency has been applied within archaeological research, it has been seen as a property held by humans alone. To have agency, one has to be able to act intentionally or consciously, in order to reach a desired outcome (Robb 2004, p. 131). As humans have been perceived by many researchers as the only entities that possess self-consciousness and the capacity for forward planning, all other non-human things (animate or inanimate) were excluded from the position of being ‘actors’ in past societies (Midgeley 1994; Philo and Wilbert 2000; Irvine 2007). Such views, based on conceptual divisions, between human/animal and culture/nature, are entrenched in Western thought. This position had led to humans being seen as organisms divided into two parts, the biological (i.e. that part shared with other animals), and the spiritual or mental, attributes unique to humans (Hamilakis 2001; Ingold 1995). Perceived as being devoid of thoughts, objects and animals have therefore been frequently ascribed passivity, seen as material things which are used at will by conscious human actors to impact on their relationships with other humans (Robb 2010, p. 502). The first move away from such a view of agency was put forward by (Gell 1998, p. 17). He argued that non-humans can be seen as agents if they are believed to initiate an event and humans attribute intentions to these occurrences. Irrespective of whether non-humans actually did act intentionally or not (see below), ‘agency’, in this sense can be seen as a culturally defined position. Put another way, an agent is whatever a human, within a particular social context, thinks it is. Humans and non-humans are not, however, given equal weight within this framework, hence, Gell’s use of the terms of ‘primary’ and ‘secondary’ agents for the former and the latter. Although he stressed the relational and contextual nature of agency, the concept of intention (attributed or otherwise) remained paramount.

Recently, however, archaeological studies have drawn on the work of social theorists, such as Callon (1986), Latour (2005) and Law and Mol (1995), which emphasise agency as a relational idea, for which intention is not a prerequisite. Rather than humans or non-humans acting alone, social contexts and meanings are generated through engagements between people and the material world around them (Latour 2005, p. 80). Accordingly, within the framework of associations between the varied aspects of the material world, non-humans can also be said to have agency (Law and Mol 1995, pp. 276-277). Recognising that humans are not the only ones with agency means that, in our interpretations, we must apply the same level of attention, standards and values to the respective actors (human or otherwise) within social contexts (Shanks 2007, p. 591). Material things, after all, have their own intrinsic qualities that will constrain how they can be produced or obtained, how a person might use them, pass them on and so on (DeMarrais *et al*. 1996). As interactions can vary and the material world can be used in diverse ways, categories are fluid and the same agent can hold a range of different, or a ‘patchwork’, of meanings (Law and Mol 1995, pp. 290-291; Murdoch 1997, p. 741). This contrasts with the processes typically involved in artefactual analysis, whereby objects are fitted into typologies, thus giving the impression that these objects were seen in a fixed, universal way (Jervis 2011, p. 241). Whilst this is a necessary step in making sense of archaeological remains, comprehending the diverse ways in which humans and non-humans were categorised and gave each other meaning, requires looking at the actual engagements that took place between them.

Despite the use of relational approaches by archaeologists in order to remove ontological divisions and recognise the potential agency of non-humans (Murdoch 1997; Witmore 2007), attention has predominantly focused on studies of artefacts and technological processes. This was a point also raised by Jones and Cloke (2008, p. 86), who noted that “organic non-human others…are sometimes confusingly absent” within research. Some social scientists have recognised the agency of living beings, namely animals, in their work (e.g. Birke *et al*. 2004; Haraway 1992; Law and Mol 2008; Philo and Wilbert 2000). Such studies have demonstrated the capacity of animals to affect humans and their environment and to act against the wishes of people. This is because, when we encounter animals, we are involved in true *interactions*, rather than just the *actions* imposed by people on things, subject to their material properties (Argent 2010; Reed 1994, p. 112; Soderberg 2004, p. 167–168). Yet, archaeologists still frequently seem reluctant to include animals within their reconstructions of social contexts, beyond their roles as sources of food and labour; leading to an unsatisfactory position in which inanimate objects are seen to act, but animate beings are not. This paper seeks to address this situation, by demonstrating how and why animals in the past should be viewed as agents, and that human–animal engagements could be of greater importance in creating the social and identity than human–object actions. It will be argued that, rather than being relegated to the periphery of discussions of the social, animals should be central to them. Moreover, we need to recognise that the category of ‘animal’ actually contains a variety of species, with different behaviours, appearances and varying degrees of proximity to humans. Accordingly, we must reconstruct relations on a species and, often, an individual level. In order to do this, this paper follows Birke *et al*.’s (2004) application of performativity theory, as reformulated by Barad (2003), to human–animal relationships. Performativity, or what things actually *do*, is the process by which relations between humans and non-humans, categories and meanings are enacted into being (Law 2009, p. 150–151; Law and Mol 2008, p. 74). Rather than human and animal identities being static, this paper takes the position that these aspects were instead dynamic, depending on the context and the nature of interaction.

The focus of this paper is the domestic cat, *Felis catus*. As a species with a close but frequently contradictory relationship with humans, it is ideal for exploring the agency of human–animal interactions. Anglo-Saxon England is the time and place that will be analysed, as it witnessed a range of social developments, including the end of Roman centralised control, widespread immigration, increasing social hierarchy, the spread of Christianity and the development of towns (see below). These processes impacted upon and were driven by the material world, particularly human–animal links, so that understanding such interactions gives us a better understanding of society in this period (Poole Forthcoming a, b). Additionally, a wealth of sources survive from this time, which enable us to perceive what these interactions consisted of, including zooarchaeology, law codes and poetry, which can be combined with behavioural ecology and cognitive studies. Given the complex and oft-contradictory nature of human–animal interactions, obtaining the most holistic perspective of such relationships requires us to integrate as many sources as possible. Moreover, drawing upon varying sources of evidence can help to counteract the deficiencies of some types of data. Bones themselves are an excellent source, because they are the physical residue of past encounters between humans and animals, although the treatment of an animal in death may not always represent how it was perceived in life (see below). Textual sources provide some information on how cats were treated and categorised in life, but given that they derive from elites and ecclesiasts, may not be representative of the wider population. Due to the nature of these sources, it is possible that they emphasise some facets of human–cat relations, such as how they were used, more than other aspects, such as affectionate ties between parties. These limitations of the data must be borne in mind throughout the discussion here. However, before considering cats, attention will focus on how animate beings differ from inanimate objects and the conceptual challenges this presents archaeologists.

## Animals as Agents

Whilst some archaeologists have argued that animals can be seen as material culture (e.g. Pluskowski 2007), they actually differ from objects in important ways. Animals can certainly be culturally appropriated and used to structure social action (Jones 1998, p. 302). Domestic species, for example, have reached their modern forms through a process of selective breeding to meet human needs (Clutton-Brock 1999). However, animals are not simply passive beings, as, like humans, they are biological organisms, with their own objectives and motivations, even though we may not understand these (O’Connor 2007a, p. 9). Many traits of animal breeds can simply be side effects of human control over breeding, and others may only emerge after a long period of time (Zeder *et al*. 2006, p. 4). People do not literally *make* animals, but rather establish the conditions for the growth and development of their livestock (Ingold 1996, p. 21). The key difference between animals and objects, however, is that animals are aware of their surroundings (even if their sensory perception may be different than humans), and they are capable of acting upon them (Gibson 1979; Reed 1994, p. 116). In this way, animals, like humans, can be seen as ‘action centres’ (Heider 1958, p. 21). They are perceived differently to objects, as they can physically intervene in a situation without the involvement of others. For example, a gun used by a human can lead to another person being shot, but a dog does not need to be made to bite a person. They have the potential to do so themselves. This affects human perception of animals, and in fact, humans distinguish between animate and inanimate beings from a very early age (Gelman and Opfer 2002). A wide range of animals are also capable of differentiating in the same manner, including distinguishing between different kinds of animate things, such as predator and prey (Reed 1994, pp. 110–111). Although humans can learn to recognise the behaviour and attitude of other living creatures, their unpredictability affects how we interact with them. As a contemporary herding manual suggests, for an animal herder, the first lesson that must be learnt is that “[y]ou aren’t going to get to do it the way you want” (Cote 2004, p. 9, cited by Mlekuž 2013, pp. 155–156).

For example, milking an animal is not just a case of extracting the milk by force. It involves close, physical contact, which plays an important role in establishing emotional bonds with that animal. In developing such relations of mutual trust, the process of milking can be more pleasant, safer, and as it can increase milk production, ultimately more profitable (Bock *et al*. 2007, p. 112). The same would have been true of animals in the past as well (Poole 2013a), with people, whether farmers or not, organising their way of life, landscape and diet according to the animals inside and outside of their care. The question of whether or not animals resist or co-operate with humans due to intentions, instincts or a combination of the two is a difficult one to answer. As Midgeley (1994, p. 38) has argued, the influence of Cartesian thought, in particular, has led to animal behaviour frequently being perceived in terms of instincts, with animals reacting through innate programming. Although they exist, they are seen to have no sense of themselves as individual entities, capable of relating to the environment around them, something which is a major requirement for having a sense of ‘self’ (Mead 1962; Wood-Gush *et al*. 1981, p. 46). There is now a great deal of evidence that many species of animals, including apes, dolphins, monkeys, cats and dogs, do have a sense of ‘self’; they are capable of adapting their behaviour, which implies consciousness because it suggests they monitor their *own* actions (Irvine 2007). This does not mean that animals have the same self-awareness or ability to plan for the future as humans do. Yet these animals have the capacity to act in this way, at least to some degree, meaning that a sense of self-awareness is not the sole preserve of humans. Moreover, within the context of relational approaches, it is not necessarily important whether one accepts that animals act intentionally or have a sense of self—what is significant is that they have the capacity to shape their world (Philo and Wilbert 2000; Fudge 2006; Law and Mol 2008). Excluding animals from reconstructions of the past thus removes a significant, real, presence in peoples’ lives, whilst animals’ potential to act back creates more dynamic and uncertain relationships than those between people and objects.

Nonetheless, as with objects, the characteristics of animals also affect what we might choose to do with them and the response we receive. Objects do not have static meanings, but rather can be potentially perceived and categorised in diverse ways, depending on how they relate to humans and other material elements (Jervis 2011, pp. 240–241). The same is also true for animals. As Law and Mol (2008) demonstrated in their study of sheep in Cumbria, England, during the foot and mouth epidemic, these animals were simultaneously categorised as veterinary sheep, epidemiological sheep, economic sheep and farming sheep, amongst other roles. Each practice presented a variation on ‘sheep’ thus creating a ‘sheep multiple’, one animal (or group of animals) which could be assigned to different, but interlinked, categories (Law and Mol 2008, pp. 65–66). Whilst notionally a sheep belongs to a particular species, they could be perceived in a number of different ways. O’Connor (2010, p. 271) highlights the problems of the domestic/wild opposition in archaeological research. He argues that this dichotomy categorises the *species*, rather than the *relationships* between humans and animals. It is necessary to realise that attributing animal bones to various categories such as ‘cattle’, or ‘sheep’, or reconstructing mortality patterns, is not the point at which interpretation stops, but rather where it should begin (Serjeantson 2000). Whilst domestic/wild categories can be useful for some analyses, understanding the relations between people and animals therefore involves attempting to reconstruct the varied interactions that took place between them in life (and hence the meanings attached to them within the overall existing ‘patchwork’) (Knight 2005, p. 5). Not only does this involve comparing differences between how various species were related to, but even of the associations between people and individual members of a species. In seeking to understand these relations, the notion of performativity is a useful interpretative tool.

Performativity has, in the past, been used to emphasise the ways in which speech and gestures perform identities and meanings and is most widely known through Butler’s (1993) study of gender and sexuality. Contrasting with such approaches to performativity, which place emphasis on language and signification, Barad’s (2003) concept of ‘agential realism’ foregrounds materiality in the process of creating meanings. For her, the world consists of phenomena, which are produced through the *intra*-*action* between components of the world. Barad (2003) coined the term ‘intra-action’ to replace ‘inter-action’ in order to highlight that agencies do not precede encounters, but rather that agency emerges from the relationships between components. Importantly for the purposes of this paper, Barad (2003, p. 817) also points out that, “[a]gential intra-actions are specific causal material enactments that may or may not involve humans”. It is this grounding of agency within materiality and the inclusion of non-human entities that make this form of performativity especially helpful for our purposes. This is shown by Birke *et al*. (2004), who adopted it to demonstrate how notions of humanity and animality are not natural states, but are instead performed. They show how the ‘laboratory rat’, far from being a static entity, is in fact co-created through the practices in which these animals are involved. These rats are specifically bred to be kept in certain areas of laboratory space and utilised for particular activities, while a whole range of equipment has been developed to fit them. Importantly, however, rats are not passive objects in this; scientists have to rely upon them to breed, whilst a rat also has the capacity to squeal or bite the person handling it. Although potentially unaware of it, the laboratory rat actively participates in the creation in meaning and the way they are categorised. This situation creates a category of animal far removed from its counterpart in the wild. Accordingly, what we term the laboratory rat is to some extent a hybrid, comprised of intra-action between animal, people and related technologies (Birke *et al*. 2004, p. 173). Within this context, the rat, through their actions, is also being acted upon; they are, as Law and Mol (2008) put it, ‘the actor-enacted’. Yet in order to understand *how* particular animals affected humans in the past (and vice versa), we first need to understand the animals themselves and how they engage with others (Argent 2010, p. 161; Birke *et al*. 2004, p. 174).

## Cats Themselves

The domestic cat, *F. catus*, is at present seen by most zoologists as a separate species from its supposed ancestor, the wildcat (*Felis silvestris*) (Kitchener and O’Connor 2010, p. 83). Wildcats and domestic cats show a great degree of behavioural flexibility and the degree of contact between members of the same species and the make-up of social groups varies, depending on the sex of the animal and the availability of food resources (Pontier and Natoli 1996, p. 85). Even wildcats, which are frequently seen as solitary beings, show adaptability in their home range, with a high amount of overlap if there is a concentration of food and mates in an area (Liberg and Sandell 1988). The focus of scavenging opportunities and prey species (such as rodents) created by human settlements are seen as having created ecological conditions which favoured selection for greater tolerance between wildcats than when food resources are more widely dispersed. The process of adapting their own behaviour to each other and becoming tolerant of human company was thus central to the development of the domestic cat (Crowell-Davis *et al*. 2004). This illustrates that, rather than humans imposing domestication onto passive things, the choice of cats to associate themselves with humans was paramount; as Reed (1980, p. 16) points out, “We did not domesticate the cat so much as the cat became tolerant of, and adopted us”. Within the cat colonies that form, females provide the basis of the social group, which is organised along matrilineal lines (Crowell-Davis *et al*. 2004; Liberg and Sandell 1988). Male cats are more likely to be aggressive to other cats, especially those of the same sex, particularly when in the presence of a fertile female, although this is not always the case (Lindell *et al*. 1997).

Such variability in cat behaviour is a useful reminder that a ‘universal cat’ does not exist, either now or in the past (Bradshaw 2013, p. xv). This contrasts with objects, such as a particular type of pottery, which might have slight variations or be used in different contexts and thus be categorised in varying ways (e.g. Jervis 2011) but whose essential properties, such as fabric and form, tend to be the same for individual pots of the same type. Domestic cats, whilst belonging to the same species and possessing similarities in morphology and general behavioural characteristics, can actually vary considerably in their temperament and actions towards humans. The latter aspect is determined by a complex interplay of genetic factors and personal experiences (Bradshaw 2013, Ch. 4; Mendl and Harcourt 2000). Many modern cat breeders, interested in the friendliness of cats towards humans, select breeding cats with this in mind. This situation is perhaps why there is potential variability in behaviour, depending on the particular breed to which a cat belongs (Mendl and Harcourt 2000, p. 52). Domestic cats exhibit a wide variety of coat colours and patterns, in contrast to the tabby-like appearance of wildcats (Kitchener and O’Connor 2010, p. 87). It is not clear, however, to what extent people in the Anglo-Saxon period were capable of, or interested in, controlling the breeding of cats. As recently as the Victorian era, Charles Darwin noted that ‘cats, from their nocturnal rambling habits, cannot be matched and…we hardly ever see a breed kept up’ (*On the Origins of Species*; Bynum 2009, p. 47). The existence of distinct breeds in Anglo-Saxon England thus appears unlikely. In terms of a cat’s learning experiences, not only are the type of interactions they have with humans important for determining how they relate to people, but the timing of this interaction is also vital. The ‘socialisation period’, a time when social contact with humans is required to begin, to prevent cats becoming fearful towards them, only lasts between weeks 2 and 7 of a cat’s life (Karsh and Turner 1998). The greater the level of interaction (assuming it was positive) between human and cat during this period, the more friendly the adult cat is likely to be. However, cats continue to learn much more about how to interact with people throughout the first year of life (Bradshaw 2013, p. 107). Importantly, cats are only second to dogs in their ability to discover how to act towards their own species and humans, almost simultaneously, and both are much more adaptable than other domestic animals at doing so (Bradshaw 2013, p. 101).

How people in Anglo-Saxon England understood the interaction between cats is unclear. Knowledge of human–cat interactions in this period is unlikely to have been as in-depth as modern studies currently allow. Nonetheless, some of the behavioural traits would have been more readily apparent than others, whilst interpretation of these traits may well have differed from our own. Cats have certain *affordances*, to use Gibson’s (1979) term, which constitute their inherent biological features in relation to human sensory capabilities. From a human perspective, we can determine that cats are living beings, and we can see them, hear them, see them, smell them, touch them, but we may not be able to understand the full range of cat communication. For example, we do not have the same level of eyesight in darkness as cats, our hearing is not as acute, nor is our sense of smell. This means that much of the information shared between other cats (and perhaps other non-human animals) is missed (Bradshaw 2013, Ch. 5). Nonetheless, humans may identify the affordances of an object or animal in relation to the possibility of their use for particular actions. For example, a cat is good for many purposes, as will shortly be discussed, but it is clearly unsuitable for tasks such as pulling a cart or herding sheep. Yet, we cannot just focus on intrinsic qualities of things, as this gives little room for considering the impact that the ongoing interaction between person and non-human might have on perception. For this reason, the concept of *affect*/*affection* was adopted by Lorimer (2007), in his discussion of non-human charisma, because it is more concerned with the *process* of perception and generation of meanings, through the embodied meetings between entities. Both the affordances and affects of cats would have been important in encounters between them and humans throughout the Anglo-Saxon period. Returning to Birke *et al*. (2004), what cats meant, and the various sub-categories to which they belonged, were determined by performativity, as will now be discussed.

## Anglo-Saxon England and the Physical Remains of Cats

Anglo-Saxon England (*c*. ad 410–1066) represents a time and place that underwent considerable social, economic, political and religious change (Hamerow *et al*. 2011, which this paragraph draws from). The Early Saxon period (*c*. ad 410–650) was characterised by migrations from other parts of northern Europe, including northern Germany, the Low Countries and southern Scandinavia. In some cases, existing populations were displaced, whereas in others, they lived alongside the newcomers. Despite the influx, there may have been some population decline which, joined with the end of Roman centralised control, urbanism and the villa economy, created less pressure on food resources. Farming was smaller-scale and focused on local needs. Emphasis became placed on portable wealth, especially domestic livestock, with cattle being particularly prized. Over the course of this period, society became increasingly stratified, with the development of a series of territories controlled by leaders, which eventually became kingdoms. In ad 597, the Augustinian mission arrived, beginning the process of reintroducing Christianity to many areas of the country, which continued into the Middle Saxon period (*c*. ad 650–850). Changes in burial practices, as well as Church writings and condemnation of certain practices, indicate that Church leaders imposed ideological boundaries between humans and animals, in contrast to more fluid perceptions in the Early Saxon period. The spread of Christianity was assisted by the increasing power of some elites, with many kingdoms being consumed by others, leading to larger kingdoms and increasing social hierarchy. Whilst portable wealth was still important, from this period onwards, growing emphasis was attached to land ownership. Nonetheless, desire for high-status items was a major factor in the development of settlements known as *wics*, sites of an urban character, which became centres for craft production and trade. However, these sites began to decline, in part due to the impact of raids by Scandinavians, although in the Late Saxon period (*c*. ad 850–1066), the focus of these peoples was on securing territory and settling. This led to destruction of kingdoms and the control of a large area of the country by people of Scandinavian origins. Despite such events, this period saw the emergence of urban centres, increasing power of the Church and the gradual reclamation of England into one kingdom, before the Norman Conquest in ad 1066 brought the period to an end.

During this epoch, there were three feline species present, the domestic cat, the wildcat and the lynx. However, the latter species, whose bones can be confidently distinguished from the other species, is only known from one specimen recovered at Kinsey Cave, Yorkshire, and radiocarbon dated to ad 425–600 (Hetherington *et al*. 2006). Wildcats are part of England’s native fauna but do not appear to have been the ancestors of domestic cats in this country. Instead, domesticated cats seem to have brought in by humans, at least during the Iron Age, but possibly as early as the Neolithic (Kitchener and O’Connor 2010, pp. 90–96). During the timeframe in which wildcats and domestic cats have co-existed in Britain, including Anglo-Saxon times, it is likely that at least some interbreeding between them took place. This was potentially a more regular occurrence in more isolated, rural, settlements given the wildcats’ aversion to open habitat and avoidance of humans (Kitchener and O’Connor 2010, p. 85). Morphologically, wildcats are generally larger than domestic cats, although there is a size overlap between male domestic cats and female wildcats. This, coupled with the possible existence of domestic–wildcat hybrids, can make it difficult to differentiate between their bones within archaeological assemblages, rendering their overall distribution in Anglo-Saxon England unclear. However, O’Connor (2007b), using a log-ratio metrical technique, has tentatively identified wildcat bones from Late-Saxon Coppergate, York and Lincoln, although it is most likely that these animals came from outside the towns, or perhaps were even traded from elsewhere. To date, these are the only sites in England from this period which have been suggested to have wild cat remains, although this may be at least in part to researchers not attempting to make the distinction within their bone assemblages. This paper assumes the majority of cat bones recovered from Anglo-Saxon sites come from ‘domestic’ cats. This categorisation of cats fits with contemporary textual evidence. The Old English term for cat was *catt* or *catte*; whilst of uncertain etymology, it may stem from the Latin *cattus*, a term which Isidore of Seville says derives from catching (*captura*) (XII.ii.38; Barney *et al*. 2006, p. 254). If the term was of Latin origin, it is uncertain whether the meaning was known in Anglo-Saxon England, although as we shall see, being a mouser was often a significant role for cats in this period. Beyond this basic distinction, a cat would have been classified in a range of different ways, depending on what they did, how people interacted with them and so on. Understanding what the relationships were will thus help to inform us about how cats were categorised.

With regards to the links between people and animals in the past, their physical remains—mostly in the form of their bones—provide vital insights. Aspects such as species ratios, animal ages at death and butchery data are often used to research economic aspects of past societies, but these tend to represent the end products of human-animal relationships. However, such information can also reveal a considerable amount concerning their contact with humans in life. Given their central role in creating social worlds, these interactions were at least as important as the end result (Poole 2013a, b). Reconstructing past human–animal relationships is not without its challenges, as some species may be less frequently recovered in excavations. This alone may say something about the extent and level, or type, of interaction. The complex, multi-directional, nature of human–animal relationships necessitates an integrated approach, with evidence here also drawn from documentary sources, anthropology and behavioural ecology. In Anglo-Saxon England, the vast majority of the population was involved in food production, and animals were central to everyday life, if not more so. However, as with other societies, the types of animals that people interacted with, and the level of engagement, would have heavily depended on human social identity, including status, gender and religious affiliation (Poole 2013a, b, c). Such factors would have operated at a broad social level, although there may have been individual exceptions to these rules, with some people engaging more with particular animals, such as cats, due to personal preference. Certain animals, such as cattle, sheep and pigs, would have been encountered regularly in some form, as indicated by the frequency with which their remains are recovered from sites of this period (Table 1). Others, including those animals we might label as ‘wild,’ would have tended to be more socially distant, although the level of interaction would have depended heavily on species. Cats throughout this period appear to have existed somewhere in between these perspectives.

**Table 1:** Relative frequencies of domestic mammals, as a percentage of the Number of Identified Specimens (NISP), by period and site type

Phase	% cattle/sheep/pig	% horse	% dog	% cat	Total
Early Saxon total	96.8 %	2.1 %	0.8 %	0.3 %	3.2 %
Mid-Saxon rural	94.1 %	4.2 %	1.2 %	0.3 %	5.9 %
Mid-Saxon urban	99.1 %	0.3 %	0.2 %	0.4 %	0.9 %
Mid-Saxon ecclesiastical	92.6 %	3.6 %	3.3 %	0.5 %	7.4 %
Mid-Saxon high-status	97.7 %	1.8 %	0.3 %	0.2 %	2.3 %
Mid-Saxon total	98.1 %	1.2 %	0.4 %	0.3 %	1.9 %
Late Saxon rural	90.7 %	5.1 %	3.5 %	0.7 %	9.3 %
Late Saxon urban	97.7 %	1.2 %	0.8 %	0.8 %	2.3 %
Late Saxon ecclesiastical	95.4 %	3.9 %	0.4 %	0.3 %	4.6 %
Late Saxon high status	96.2 %	2.9 %	0.6 %	0.3 %	3.8 %
Late Saxon total	95.1 %	3.3 %	1.3 %	0.3 %	4.9 %

Cat bones make up a relatively small proportion of faunal assemblages recovered from Anglo-Saxon settlements, averaging from 0.2–0.8 % of the remains, compared with other domestic species (Table 1).^1^ Nonetheless, they are regularly recovered during excavations of these sites (Table 2), with some variation by site-type. Cats are marginally better represented, as a percentage of the Number of Identified Specimens (NISP), on urban rather than rural sites. They are found on 41.7 % (10 of 24) and 88.2 % (15 of 17) of Mid-Saxon rural and urban sites respectively, and in the Late Saxon period, on 53.8 % (7 of 13) of rural, and 77.8 % (35 of 45) of urban sites. Even on those sites where their bones have not been found, this does not necessarily mean that cats were absent from the vicinity; the small size of cat bones means that they are very vulnerable to poor recovery or rapid excavation, meaning that their bones may be missed (Kitchener and O’Connor 2010, p. 91). Nonetheless, this makes ecological sense, as the greater concentration of people in towns is likely to have led to a larger/more concentrated food supply at such settlements than at rural settlements, making them able to support larger cat populations. Their reliance on human waste in towns raises the interesting prospect that cat populations significantly suffered with the decline of towns towards the end of the Roman period and into the Early Saxon era (Engels 2001, p. 139). One of these food sources may have been rodents, which were also attracted to towns by the accumulation of food there. It is thus also possible that cats maintained greater numbers in towns because people kept them as mousers, or perhaps as pets (see below). The Anglo-Saxon evidence indicates that cats were more widespread on Middle and Late Saxon sites than in the Early Saxon period (Table 2). This may partly relate to the potential for greater numbers of cats created by towns, which did not exist in the Early Saxon period. However, when we look at relative proportion of the domestic mammal remains, there is no variation when we take the total figures for each period.

**Table 2:** Frequency with which cat remains have been identified from zooarchaeological assemblages, by period and site type

Phase	No. sites	No. with cats	% of sites with cats
Early Saxon rural	36	18	50.0 %
Mid-Saxon rural	24	10	41.7 %
Mid-Saxon urban	17	15	88.2 %
Mid-Saxon ecclesiastical	4	4	100.0 %
Mid-Saxon high-status	10	7	70.0 %
Mid-Saxon total	55	36	65.5 %
Late Saxon rural	13	7	53.8 %
Late Saxon urban	45	35	77.8 %
Late Saxon ecclesiastical	3	3	100.0 %
Late Saxon high status	13	9	69.2 %
Late Saxon total	74	52	72.2 %

Even so, it is probable that cats were present around the great majority of settlements throughout this period, albeit in smaller numbers than typical ‘food’ species, such as cattle, sheep and pigs. The relationship between cat and human was thus more likely to have been one-on-one, as opposed to other animals, such as sheep which might well have been interacted with individually but which were typically part of a flock (on rural sites at least). A further measure of cat–human interactions is apparent when considering other aspects of the faunal data, especially if we take into account differences between site types. It is difficult to quantify the ageing and butchery evidence for cats in Anglo-Saxon England, due to the sometimes patchy nature of reporting, and the varying ageing methods used by different researchers. However, some useful information can be identified. At Early Saxon West Stow (Crabtree 1989), out of 274 cat bones, two had cut marks on mandibles, one pelvis had skinning marks, and one atlas vertebra had a cut, thought to derive from slitting the animal’s throat. For the Middle-Saxon period, the only butchered cat remains described were from a high-status phase at Eynsham (2b), Oxfordshire (Mulville 2003), where one of three cat bones had cut marks and the high-status site of Bishopstone (Poole 2010). In contrast, none of the cat remains from rural Mid–Late Saxon Sedgeford (Poole unpublished report) or Mid-Saxon urban sites are reported to have butchery marks. None of the reports dealing with Late Saxon faunal assemblages indicate cat butchery in a rural context (although the sample size is relatively small), whilst a number of contemporary urban assemblages do: Castle Mall (Albarella *et al*. 2009) and Norwich Cathedral Refectory (Curl 2006), Norwich; Coppergate (O’Connor 1989) and St Saviourgate, York (Carrott *et al*. unpublished report); St Ebbe’s, Oxford (Wilson 1989) and Winchester Staple Gardens (Lena Strid, pers. comm.). At Coppergate (O’Connor 1989), there were also groups of cat phalanges apparently divorced from the rest of the cat, indicative of skins. It would therefore seem that there was at least some commercial exploitation of cat furs in towns, although exactly how extensive this was is uncertain. Notably, none of the cut marks on cat bones from this period indicate that the cat was seen as a food source, which fits with documentary sources that portray them as a last-resort food (Hagen 1995, p. 187). The ageing information is even harder to assess than the butchery evidence (Table 3). Often, cat ages are only mentioned when they are represented by partial or complete skeletons (also known as Associated Bone Groups, or ABGs; Morris 2011). The primary ageing data for cats are the state of fusion of their bones and dental eruption data. A wide range of ages are evident, from very young to adult, with a tendency for cats recovered from urban sites to be younger. A few site reports also mention instances of cat pathology, which may provide some insight into how cats were treated (Table 4). We must bear in mind that these constitute a very small number of the total bones, and we also need to allow for the existence of soft tissue injuries/disease, which have not affected the bone, and so are invisible to the zooarchaeologist. Nonetheless, of the small sample, all four cases seem to have been the result of some sort of trauma.

**Table 3:** Ageing data for cats, by period and site type

Site	Site type	Period	Ageing
Walton, Aylesbury	Rural	ESAX	All bones unfused
Cadley Road, Collingbourne Ducis	Rural	MSAX	Two adult cats
Kings Meadow Lane, Higham Ferrers	Rural	MSAX	Subadult
Maxey	Rural	MSAX	One young cat
Sedgeford	Rural	MSAX	Mixture of adult and immature cats, but mostly adult
28-31 James St, London	Urban	MSAX	Adult humerus, ulna, tibia and one immature ulna
National Portrait Gallery, London	Urban	MSAX	One mandible from cat over 5–6 months old
Peabody/National Gallery, London	Urban	MSAX	40 % of bones are from juveniles
Melbourne Street, Southampton	Urban	MSAX	4 bones >1 year, 11 > 8.5 m, 4 younger than this, 3 kittens also present
Bishopstone	High status	MSAX	Several cat ABGs, ranging in age from >5–6 m to adult
Lake End Road, Dorney	High status	MSAX	Three adult cat bones—two mandibles with permanent teeth and one fused distal tibia. One immature (unfused proximal radius)
Bishopstone	High status	M/LSAX	Several cat partial and complete skeletons, ranging in age from >5–6 m to adult
Ashwell Site, West Fen Road, Ely	Rural	LSAX	Of two cat skeletons, one was adult and one a kitten
Sedgeford	Rural	LSAX	Mixture of adult and immature cats, but mostly adult
Castle Mall, Norwich	Urban	LSAX	High percentage of unfused bones
The Green, Northampton	Urban	LSAX	Of three cat skeletons, one was well-grown but immature (also missing the head)
Lower High Street, Southampton	Urban	LSAX	Majority of cats are not adult; only two of 14 latest fusing bones were fused
Winchester Staple Gardens	Urban	LSAX	Several skeletons of adult cats and kittens, only two to three bones from mature animals, the remainder from kittens
Wraysbury	High status	LSAX	One cat was less than 1 year old

**Table 4:** Sites with cat pathological evidence, with details/interpretation of pathology

Site	Site type	Period	Pathology/non-metric trait
Bishopstone	High status	MSAX	Humerus fractured just below halfway, but completely healed, with distal end slightly displaced medially. Slight protrusion of bone where fracture occurred.
Peabody/National Gallery, London	Urban	MSAX	Cat femur leg broken mid-shaft, badly overlapping ends; no sign of infection. Unlikely to result from a fall, but deliberate human kick, or accidental collision with passing cart.
Bury Road, Thetford	Urban	LSAX	One cat probably killed with blow to left side of braincase behind eye; same animal had a left femur shaft broken midway, with slight movement of the bone sideways, which caused bowing and heavy callus building as the fracture healed.
Flaxengate	Urban	LSAX	Fractured femur, caput and neck collapsed downwards onto shaft and fused into solid block—no apparent articular surface—cat had to keep using this: not cared for.

## Cat-egorising Animals in Anglo-Saxon England

What do these data tell us about cats in Anglo-Saxon England? The evidence for cut marks brings us to our first category of a cat—as a source of fur, presumably for clothing. This represents one particular form of engagement with this animal. Just as with modern cats, cat fur would have been available in a range of colours; the ancestors of the domestic cat were striped, and Old Irish sources suggest a variety of different coat colourings, including ginger, grey, white-breasted black cats and the famous white *Pangur Bán* (Kelly 1997, p. 123). There is no reason to believe this was not also the case for Anglo-Saxon England. As a fairly readily available product, cat skin, as well as leather and sheepskin, could have formed part of the wardrobe of lower-ranking peoples (Veale 2003, p. 4). The act of wearing cat skins was a very different relationship than interacting with, or at least being affected by, the living animal. It is not clear whether each of the cats apparently skinned were also purposefully killed or some were already dead when skinned. At West Stow, at least one of the cats seems to have had its throat slit (as indicated by cut marks on the atlas vertebra), but such evidence is not mentioned in the reports for other sites, although throat slitting may not leave traces on the bones themselves. Alternatively, if the atlas vertebrae are not recovered, this type of killing would be impossible to observe. Another method used to kill cats before making use of their skins is indicated by faunal remains from Viking-Age Odense, Denmark, where cats had their heads wrenched off at the top of the spine, leading to much of the occipital area being removed (Hatting 1990, p. 184). The interaction here was framed by a physical characteristic, or affordance, of the cat—its fur and its presence within a particular environment (mostly towns). Although the examples we have of cat skinning in Anglo-Saxon England do not occur on an industrialised scale, it is possible that some of these examples were conducted by furriers. The fact that some of the sites with evidence of processing cats for fur also have other fur-bearing mammals seems to support this.^2^ Nonetheless, in order to kill the cats, they first had to be captured, which may have been easier if dealing with cats that were used to human company than with feral cats (see below). Any cat could have run away from the advances of these people, which would frustrate the intentions of the person concerned. Even if captured, there is no reason to believe that the cat would be held and killed willingly. We can imagine the cat, like the laboratory rat, hissing and trying to scratch or bite their captor to escape. Sometimes they would have succeeded, many other times they may not have done, as indicated by the skinned cats evident on these settlements.

These human–cat interactions have not ended well for the cats concerned, but in life, cats could have been perceived and treated in a range of different ways. Key factors here are the characteristics of a cat and the effect that their behaviour had on how people perceived them. In modern society, at least, there is often a tendency to see cats as aloof, independent and somewhat indifferent to humans (Alger and Alger 1997, p. 72). Even Isidore of Seville, when describing dogs as being loyal to their masters and requiring human contact, made no such statements in relation to cats (XII.ii.26; Barney *et al*. 2006, p. 253). Although cats are capable of forming close relationships with people, they also become strongly attached to where they live, often more so than the people they live with. In contrast, dogs tend to form the closest bonds with their owners, followed by other dogs, with their physical surroundings last on their list of priorities (Bradshaw 2013, p. 209). In human–dog relations, humans have come to assume the dominant role within the dog’s social hierarchy (Clutton-Brock 1994, pp. 24–25), which relates to evolutionary history. As Bradshaw (2013, p. 193) points out, whereas dogs have worked with humans and so have needed to develop a good awareness of human body language, cats have worked independently and so required a greater awareness of their environment. This higher level of independence means that there may have been a tendency in Anglo-Saxon England, as in modern society, to see cats as more ‘wild’ than other domesticates (Griffiths *et al*. 2000, p. 58). One ‘wild’ aspect of behaviour shared by all cats would have been hunting instincts, although some cats would have felt more inclined to hunt than others (depending on the availability of food sources), whilst others could have been better hunters, depending on experiences when younger. Although predatory instincts appear to exist in all cats, practicing the relevant behaviour through activities such as ‘play’, in which the mother presents her young with live prey, can help hone the kittens’ hunting skills (Pokier and Hussey 1982, p. 136). Interaction between kitten, mother and ‘prey’ was therefore important to determining the way a cat was used and perceived by humans and the contribution that they made to fulfilling (or not) a particular social role.

The cat’s part in limiting rodent populations would have contributed to them being tolerated, during the early stages of domestication and throughout much of history, even though scientific studies indicate that their pest control capabilities may have been overemphasised (Kitchener and O’Connor 2010, p. 88). For example, the *Laws of Hywel Dda* (a tenth century Welsh source) make clear what made desirable qualities in a cat: “…that it do not devour its kittens, and that it have ears, eyes, teeth and claws, and that it be a good mouser” (Richards 1954, p. 92). Similarly, in one Old Irish legal source, a cat’s ability to purr, guard the barn, mill and corn-drying kiln from mice were esteemed qualities (Kelly 1997, p. 122). In both of the cases, the cat had a role to play. If they complied with these ‘wish lists’, then they could be highly valued, but if they did not do all of these things, or if they were a poor hunter, this would have affected how humans perceived them. In other words, being a mouser was not something that a cat *was*, it was something they *did*; they *performed* a particular role and so were labelled in a certain way. If they stopped catching rodents, or did so infrequently, then they were no longer performing such a role, despite human wishes, and might instead be acting out a different category. For those cats classed as people’s property, it is likely that, as in the Late Middle Ages, many householders deliberately refrained from feeding them, to provide incentive to hunt vermin (Thomas 1996, p. 109). The effect of such an omission is also supported by scientific studies, which suggest that while well-fed house cats kill on average 14 small animals (usually rodents) annually, feral cats kill about 1,100 small animals per year (Engels 2001, p. 1). A cat was seen as a mouser due to intra-action between human, cat and rodent; humans create settlements and foodstuffs which enable rodents to thrive, which in turn creates a population for cats to hunt, which they are encouraged to do by the withholding of food. This is a relationship that benefits cat and human. If this was not the case, the cat could choose to leave, for unlike most inanimate objects, they are capable of running away—perhaps even to visit another human, who was willing to feed them. A good mouser might have a high value, such as in the *Laws of Hywel Dda* where, if owned by the king, they were on a par with a milch sheep with her lamb and wool (Richards 1954, p. 92). This indicates that, while some of the characteristics of cats could be esteemed by people of all social levels, not all cats owned by people were perceived in the same way. The social position of their owner provided them with status connotations that other animals of the same species did not necessarily possess. This was not just a cat; they were the king’s cat, a further category within the broader designation of this animal.

It is also quite possible that cats kept as mousers were largely left to their own devices and tasks, with little human interaction. However, the same animal that acted as mouser could, at other times, or as with other cats, have fulfilled the role of a companion animal. It is difficult to identify a category of cats as pets in Anglo-Saxon England, such as we today might recognise. The term ‘pet’ has different connotations geographically and temporally, but here the definition adopted is that of ‘…animals… kept primarily for social or emotional reasons rather than for economic purposes’ (Serpell and Paul 1994, p. 129). It has been argued that Christian discomfort with close human–animal relationships would have restricted the popularity of pet-keeping for much of the medieval period (Serpell 1986; Serpell and Paul 1994). It is unclear whether the situation was different in Early-Saxon England, although it is interesting that cats are absent from graves of this period, in contrast to other domestic mammals (Bond and Worley 2006). It would seem that something in the cat’s behaviour led to its exclusion from the funerary arena, which might include its perceived ‘aloofness’ and independence from humans (see above). Nonetheless, there is some evidence from the Anglo-Saxon period, largely from contemporary societies, that close ties of affection could exist between cats and humans. This includes the ninth-century poem *Pangur Bán*, written by an Irish scholar at the monastery of St Paul in Carinthia, Austria. Here, the monk compares the work he is doing, within the scriptorium, with the work of the cat, at hunting mice. This acts as a useful reminder that functional and companionship uses of animals are not completely separate spheres. In the poem, Pangur the cat provides a useful service by hunting mice: “The job he does every day is the one for which he is fit” (Greene and O’Connor 1967, p. 83). Pangur does not seem to be a pet in the sense that we would recognise, but the fact that he has a name provides further evidence of affection, as naming animals serves to recognise an individual in their own right. Such recognition only develops due to relations that take place between humans and individual animals (Hearne 2007, Ch. 7). Moreover, when living beings are given names, this typically involves an attribution of identity that is different to places and objects (Lindstrøm 2012, pp. 155–156). Old Irish cat names such as *Méone*, a diminutive meaning ‘little meow’, as well as *Cruibne*, meaning ‘little paws’ (Kelly 1997, pp. 123–124), suggest a recognition of certain aspects of a particular individual animal’s character or behaviour. As such, these individual cats are set apart from the other, nameless and ownerless, cats. Given the long history of naming at least some animals (Lindstrøm 2012, pp. 155–156), it would seem reasonable to expect that the same was true for Anglo-Saxon England. Not all cats may have been named, but since some of them were, it suggests they were recognised as individuals, perhaps with their own ‘character’, which was highlighted and performed through interactions with humans and perhaps other cats. In this sense, the relationship is a reciprocal one, with the cat’s behaviour affecting humans enough for them to be given a name, which they may then perpetuate by continuing to act in that manner.

Regarding cats as companions, it is also important to remember that this is not only a one-way relationship; the cat benefits, in the form of shelter and warmth, and perhaps they also value the companionship. In addition to becoming more tolerant of other cats and humans as part of the domestication process (discussed above), cat communication also changed. Nicastro’s (2004) study showed that there were clear differences in the mean length and frequencies of domestic cat meows compared with wildcats, with the former being far more pleasant than the latter. This indicates that cats with more human-pleasing meows had a better chance of surviving than others. Moreover, with humans, cats are capable of modifying their meow in terms of pitch and duration, or by combining it with other sounds, to suit the circumstances. They seem to learn how to do this by testing different meows and seeing which work in particular situations, a process by which each cat and owner develop their own individual form of communication (Bradshaw 2013, p. 205). The importance of this connection between human and cat is interesting, given that (in addition to the qualities discussed above), amongst the attractive attributes of a cat within Old Irish laws was their ability to purr; if they were not able to catch vermin, they were worth half the cost, but notably, still had a value (Kelly 1997, p. 122). As cats generally display their pleasure in physical contact by purring (Serpell 1986, p. 106), this suggests a mutual pleasure in each other’s company, and actual physical contact between human and cat. Both parties here are acting together and responding to the actions of each other. The cat may well seek out this interaction by making contact with the person. Purring not only shows the cat’s pleasure, but also provides an incentive for the person to continue stroking the animal. Moreover, cats do not just possess one purr but can vary it according to what they wish to gain from a person. For example, research by McComb *et al.* (2009) demonstrated that, when cats are seeking to solicit food from humans, they adopt a low-pitch purr which contains a high frequency element evocative of a cry or meow. They appear to be taking advantage of mammalian sensitivity to cries of this nature, including those made by human infants, the frequency of which are comparable to the cat’s solicitation purr. In doing this, cats appear to draw upon sounds made when dependent on their mother, thus applying its actions with its own species to a human owner. Again, we have an example here of a cat inducing humans to act, rather than the other way round. Whilst the cat may not be exactly aware of the cause of its purr, they will no doubt learn that such a sound can result in food. Alternatively, they might not be fed, but what is important is that here the animal is clearly not a passive entity. The cat may accompany purring with kneading, especially on soft, warm surfaces, such as a person’s lap or clothes, an action that stems from kittens kneading their mothers to stimulate milk flow. As a great degree of a cat’s social actions seem to develop from mother–kitten interaction (Bradshaw 2013, p. 208), in this context, humans become almost a surrogate mother. Whilst human and cat may not realise the origin of such behaviour, the actions of stroking, kneading and so on could have resulted in bonding between them.

One wonders whether strategies such these were adopted by one of the cats at Mid–Late Saxon Bishopstone. Isotopic data from one of three cat ABGs sampled showed that this animal had a strong marine component in its diet, estimated to be somewhere between 30 % and 50 %. This is in contrast to the other cats, which had values within the normal range for a largely carnivorous animal, as well as the human remains from the same settlement, which are otherwise accommodated within the normal terrestrial range (Marshall *et al*. 2010). This dichotomy is difficult to explain, as prior to ad 1000, the consumption of marine fish was largely an elite affair (Barrett *et al*. 2004). Although this individual cat feasibly acquired this signature due to scavenging fish from middens, stable isotopes only provide an individual’s average diet over long time spans and cannot detect occasional consumption of certain foods (Müldner and Richards 2006, p. 229). Accordingly, this cat would have had to be preferentially eating fish on a regular basis, and in large amounts, for such a strong signature to occur. This suggests that this individual was being deliberately fed by someone, or a group of people, living at the site. Given that, as noted above, humans may have had little direct involvement in the feeding of mousers in order to encourage greater pest control, the fact that one cat was probably deliberately fed suggests different treatment than other cats at the site, possibly being a favoured pet. Moreover, this animal was receiving even better treatment than many of the humans living at the site. In some ways, as with the king’s cat in the *Laws of Hywel Dda*, the performance between human(s) and cat here transcends the position accorded to other cats at this time; did this particular cat belong to an elite member living at the site? As noted above, the ways in which cats can engage with humans could have played an important role in the development of positive attitudes towards them. However, from the zooarchaeological remains themselves, it is very difficult to identify animals which were well treated in life. Pathological evidence, as cited above, can suggest instances where cats were ill-treated, but it is difficult to see things the other way around. This is because we would not necessarily be able to spot prized cats in the archaeological record, as disposing of their carcasses along with waste does not necessarily indicate that they were disliked in life (O’Connor 1992, p. 112). As noted above, the relationship to a cat is likely to have differed significantly, depending on whether dealing with a live cat, a cat carcass, a cat skin and so on. This is because the capacity to interact with people is removed when a cat is no longer alive. In addition, if, as was the case in Late Saxon England, animals were not seen as having souls (Griffiths 2003, p.149), we should not necessarily expect cats to be buried as a human would have been.

Even if a cat was nominally owned by someone, their lifestyle could in fact have shared a lot of similarities with ‘feral’ cats. These were animals owned by no one but which, unlike wildcats, lived in close proximity to human settlements. Amongst feral cats, there would be differences in the extent to which they interact with humans, ranging from those living in close proximity to humans, to groups living entirely apart from people, but which were still dependent on waste produced by settlements and the vermin attracted to such sites (Griffiths *et al*. 2000, p. 58). Given the greater concentration of people living in urban centres and the resultant higher accumulations of waste compared with rural sites, feral cats may have been a particular issue for those living in towns. Although comparable modern data for feral cat mortality are lacking, a high proportion of young cats, as discussed above, would be in line with a feral population of largely uncared-for animals, suffering from food shortages, natural mortality and accidents, rather than a group of treasured pets (Kitchener and O’Connor 2010, p. 93). Whilst feral cats may have been a nuisance at times, they were likely viewed as useful, given their potential for controlling rodent populations. Even so, there are clear examples of cats acting in ways which conflicted with human desires. In some cases, the cat may be involved in the ‘theft’ of food. Irish law codes from the seventh to eighth centuries mention the recompense a cat’s owner must pay to another human if their animal had stolen their food (Kelly 1997, p. 145). Equally, in a situation familiar today, cats could defecate in unacceptable places, such as on the rushes of a floor. This was also dealt with under seventh to eighth century Irish law, with the cat owner having to compensate the landowner (Kelly 1997, p. 144). When cats urinate or defecate indoors, the main causes are that they feel anxiety and fear, usually over territorial invasion by other cats (Bradshaw 2013, p. 155–156). Given the larger numbers of cats in towns and the subsequently smaller territories available to individual cats, compared with rural areas, one wonders whether this may have been a particular issue for urban dwellers, perhaps playing a factor in how they were treated there. Another, possibly more common, problem could have been urine spraying by males, which they carry out as a means of advertising their success to females (females also spray, but rarely) and possibly to mark territory (Bradshaw and Cameron-Beaumont 2000, p. 69). This may have occurred inside or outside buildings, and the urine itself is especially pungent and unpleasant to humans, a major reason why people today usually have their male cats neutered. However, we have no evidence for such procedures being conducted in Anglo-Saxon England, and we are thus likely only dealing with male and female cats.

That male and female animal species, including cats, usually behave differently in many ways is another reason why we must make allowances for cats having individual characteristics and behaviours. Old English distinguishes between *catt* for males and *catte* for females, so that the distinction between the two appears to have been made by people. On their basic appearance, it may not have been easy to distinguish between males and females, unless one was stood next to the other, as males are generally larger than females. Variance in size difference may, however, exist between different populations of cats (O’Connor 2007b). Sexual difference could have been more readily apparent through their behaviour, so that, to some extent, male cats were seen to be those acting, or performing, in the manner of a typical male cat. Whilst both sexes may adapt how they act, depending on the situation, male cats tend to play little or no part in raising kittens and also tend to be more solitary than females. They are also known for their aggression against one another which, coupled with urine marking and their tendency to wander further than females (Deag *et al*. 2000), potentially made them less likely to form close relationships with people (although *Pangur Bán* may have been an exception to this). Female cats are much more likely to co-operate with each other, especially when this involves females from the same family. When feral cat colonies occur around sources of food, these are overwhelmingly made up of female cats, which are almost always related to each other. If food resources are large enough, the colonies may include unrelated females, but females from different families tend not to co-operate (Bradshaw 2013, pp. 164–166).

In addition to sex, the age of a cat could also have affected its performativity. The ability to grow and visibly age highlights another area where animals differ from objects. This includes growth in size, but also changes in morphology, such as the texture/patterning of their fur (Reed 1994, p. 118). Like humans, animals also act differently in their youth compared to their behaviour in adulthood. Whilst all cats potentially engage in playful behaviour, kittens are known to be especially playful. As noted above, play is an activity that prepares kittens for hunting, but also for socialising with other cats, as well as humans (Bradshaw 2013, Ch. 4). Interacting with kittens in this way could have been amusing for people in the past, as many people find it today and could have been important in forming attitudes towards this species. This may have been particularly so with human children as they too, like other animals, need to be socialised into particular ways of acting, whether with humans or animals (Birke *et al*. 2004: 175). Changes in cat behaviour as they become older may affect how people interact with them in life and, perhaps, in death. It is notable that all cat remains from Mid–Late Saxon Bishopstone with cut marks were young animals, indicating a preference for their fur, as opposed to that of older animals (Poole 2010). This seems to be the case with other sites of the period, perhaps because young cat fur was softer. Alternatively, this exploitation may have been somewhat opportunistic, with young cats killed in a bid to control feline population size, but with fur subsequently removed. Notably, at Bishopstone, there were clear differences in the ages of disarticulated and articulated remains. Of the disarticulated cat bones, 50 % of the latest fusing elements were fused, yet only one of the cat ABGs was a fully mature animal. For the remainder of cat ABGs with fusion data, one was foetal/neonatal; six were less than 7 months old whereas five were between 7 months and 1 year old. This raises the possibility of cats being perceived and treated differently, depending on their age. It suggests that younger cats were purposefully targeted and killed, possibly because they were viewed as more useful dead than alive. These cats tended to be deposited fairly quickly, whereas older cats were left exposed for some period of time, so that their bodies decomposed and became disarticulated.

The treatment of cats at Bishopstone mirrors that observed on urban sites of the Late Saxon period, suggesting similarities in the behaviour towards cats across urban and rural sites. According to McCormick and Murray (McCormick 1988; McCormick and Murray 2007), evidence from Ireland indicates an urban/rural dichotomy regarding how cats were perceived and treated in the later Early Christian period there (tenth to eleventh century ad). Based on the small sample of sites for which data was available, cats on rural sites of this period seem to have been larger (because they were better cared for and had less competition) and lived longer than their urban counterparts. Cat bones with skinning marks were also very rare on rural sites but commonly found on urban sites. These factors, it was suggested, indicate commercial exploitation of cat skins in towns and a view of cats as commodities, in contrast to rural settlements, where they were treated exclusively as pets (McCormick 1988, pp. 223–224; McCormick and Murray 2007, p. 116). Generally speaking, there are broad similarities between cat treatment on urban and rural sites in England and Ireland during the same period. This is particularly so regarding the high proportion of young cats on urban sites and the greater frequency of skinning marks on cat bones in towns. Additionally, 3 to 4 instances of cat pathology (all trauma-related) are from towns. This can be contextualised within the broader moves in Anglo-Saxon society towards urban-based craft specialisation, which affected how town dwellers interacted with the animate world, compared with rural dwellers, as well as the increasing commercialisation of transactions involving animals (Poole 2013b). However, the link between the size of a cat and the urban/rural character of a site does not seem to hold for Anglo-Saxon England, as cat size seems to vary even between different urban sites, including Late Saxon urban York and Lincoln (O’Connor 2007b). Additionally, a cat’s genetic origin will also have played a part in its final size. At Bishopstone, we see more exploitative attitudes towards many cats (but certainly not all, as shown by our well-fed example) than is evident at other rurally located sites of the period. Although occupying a rural setting, Bishopstone was densely occupied, and archaeobotanical evidence indicates that refuse accumulated on the surface, available to scavengers such as cats, before some of it was buried in pits (Ballantyne 2010). The presence of other craft activities being carried out on the site may have created a view of many cats as commodities, to be exploited for their fur. This would be comparable to the rural Irish site of Knowth, where it is only in a tenth to eleventh century phase that skinned cat bones are evident, suggested to represent industrial trading at the site (McCormick and Murray 2007, p. 50). Similar interpretations were proposed for skinning evidence from a post-ad 1000 phase at Whithorn, south-west Scotland (McCormick and Murphy 1997, p. 612). Thus, Bishopstone shared some perceptions of cats evident amongst the urban populace, indicating that, despite broad differences, the urban/rural divide was not absolute. What this evidence shows is that, whilst the activities and lifestyles carried out on a settlement could affect human perceptions of cats (as with other animals; Poole 2013b), differences in individual temperaments of cats, what people used them for, the level of interaction and so on could all have generated varying categorisations, even on an intra-site basis. The cat, therefore, could fulfil a range of rules and be seen in many different ways, yet far from being passive entities, they were key performers in the process.

## Conclusions

Object-centric approaches to past societies neglect the rich relationships that would have existed between humans and other non-human beings: animals. This is disappointing because, as noted above, the interaction, or intra-action (Barad 2003; Birke *et al*. 2004) between animate humans and non-humans created a vibrancy of relationships that extended beyond even those that could exist between human and object. Moreover, it misses the many ways in which human–animal interactions have been used in past societies in order to structure human–human relationships (Poole 2013a, b). Researchers thus need to place interactions between humans and animals at the centre of their reconstructions of previous human engagements with the material world. This does not mean that objects should be ignored, but simply that they should not be the sole concern of archaeological research. Human history is replete with instances of people adapting their lifestyles and understanding of the world according to the behaviours and characteristics of animals (Russell 2012). However, as this paper illustrates, animals also adapted their own behaviour, in order to exploit their environment, which included humans. Many of the existing analytical methods we use for studying the remains of the past (for example, zooarchaeology) provide us with the tools we need to explore how humans and animals participated in exchanges with each other. What is required is that we do not mistake the data created as the end point of our interpretation, but rather that this is the stage at which efforts at elucidating the meanings of these results should commence. For example, whilst human–animal relationships could often have a functional element to them, this was only part of the frequently complex and contradictory interactions between different combinations of these animate beings. Accordingly, studies that only emphasise human exploitation of animals, or that merely interpret animals in symbolic ways, overlook the fundamental importance of embodied encounters between human and animal in generating the social and understanding one’s place in the world.

Attempting to gain as full an understanding as possible of human–animal relationships requires an integrated approach. As noted above, any type of evidence relating to past societies has limitations, especially given the fragmentary nature of many sources. If this paper had only drawn from one source of evidence to examine human interactions with cats, we would have received only a narrow perspective. From the bones themselves, we might have formed a view of purely exploitative attitudes being present towards cats, based on their young ages, the skinning marks and occasional evidence of trauma. From the textual sources, we see cats being used as mousers, as well as the possibility of affectionate relationships existing, based on the allocation of names to cats. The provision of names, when combined with isotopic data, emphasises that people did not so much interact with cats as a species, but as individuals, with their own personalities. Combining sources can therefore, to some extent, make up for the deficiencies in others, enabling us to build up as holistic a view as possible of human–animal relationships. This is especially important, given the diversity of animals and means by which people may interact with them; as shown by the example of cats in the Anglo-Saxon period, although nominally one species, they could be categorised and treated in numerous ways. Wild, domestic, feral, young, old, pet, nuisance and valuable are just some of the adjectives that could be linked with the early medieval ‘cat’. Following Law and Mol’s (2008) description of Cumbrian sheep, we can therefore also talk of the ‘cat multiple’; one species that could consecutively, or simultaneously, belong to a number of different categories. Such identities were in part determined by the physical characteristics and capabilities of cats, but were not static, or simply imposed by humans. Since the earliest stages of cat domestication, through to Anglo-Saxon England and beyond, the idea of a cat, and their many varied roles, resulted from intra-action between the cats themselves, humans, prey species such as rodents and the settings within which these encounters took place. Where a cat differed from an object is that they could run away from people, or seek them out for attention, could do as a person wanted, or could explicitly do what *they* wanted, even if this was against the wishes of their owner. In other words, they could directly intervene in human affairs and, as a living creature, would have been viewed very differently than an inanimate object (see also Poole 2013a). Although animals may not have the same level of conscious thought or complexity of language as humans (Coy 1994, p. 77), the key issue is that they were ‘action centres’, literally capable of *interacting* with people. Cats had perspectives on humans, even if we may not be able to completely understand what these were. When a cat was killed or died, its ability to impinge on human thoughts and emotions was dramatically affected. They could become an item of clothing, if exploited for fur—in such a case, this might still be recognisable as deriving from a cat, but this was in a very different configuration than the live animal. The diversity of interactions with animals make performativity theory, with its emphasis on this ongoing nature of identity and social meaning, an especially valuable interpretative tool. Although attention in this paper has focused on just one species, in one era, such an approach would help us to better comprehend the impact of the interplay between humans and a wide diversity of species, in a myriad of periods and places.

## Footnotes

1 Poole, K. (2011, unpublished). The nature of society in England, *c*. ad 410–1066. University of Nottingham: Unpublished PhD Thesis. Data were compiled through personal analysis of animal bones from Bishopstone (East Sussex) and Sedgeford (Norfolk) (Poole 2010; unpublished), combined with data from published excavation reports, along with unpublished data. The latter were accessed through contact with zooarchaeologists, either directly, or through the ZOOARCH mailing list, as well as through access to the library at English Heritage, Fort Cumberland. In total, 183 different assemblages were of sufficient size for inclusion in this study, of which 37, 47 and 48 assemblages could be attributed specifically to the early, middle and late Anglo-Saxon periods, respectively.

2 These are: otter, polecat and bear (the latter probably representing the importation of a skin from another country) at Coppergate (O’Connor 1989); fox and ferret at Winchester Staple Gardens (Lena Strid, pers. comm.); fox at St Ebbe’s, Oxford (Wilson 1989). In addition, in Saxo-Norman (tenth–twelfth century) deposits at Hertford, Hertfordshire, a wide range of fur-bearing mammals were represented, including domestic cat, pine marten, otter, red squirrel, hare, mole and at least three partial skeletons of red fox, suggested to represent activities of furriers or fellmongers within the town at that time (Baxter and Hamilton-Dyer 2003).
